# Comparison of sleep quality in peritoneal dialysis and hemodialysis patients: A cross-sectional study

**DOI:** 10.12669/pjms.41.8.12090

**Published:** 2025-08

**Authors:** Ergun Parmaksiz, Elif Torun Parmaksiz

**Affiliations:** 1 Ergun Parmaksiz Associate Professor, Department of Nephrology, Health Sciences University, Kartal Dr. Lutfi Kırdar City Hospital, Istanbul Turkiye; 2 Elif Torun Parmaksiz Department of Chest diseases, Health Sciences University, Sancaktepe Ilhan Varank Training Hospital, Istanbul Turkiye

**Keywords:** Sleep quality, Dialysis, Pittsburgh Sleep Quality Index

## Abstract

**Objective:**

Sleep quality is a key component of quality of life. In dialysis patients, it can be significantly affected by physical and emotional stress. Many dialysis patients experience sleep disturbances. This study aimed to evaluate sleep quality in dialysis patients and compare it between those undergoing peritoneal dialysis (PD) and hemodialysis (HD).

**Methodology:**

This observational, cross-sectional study compared sleep quality in end-stage renal disease (ESRD) patients based on dialysis modality. Data were collected from adult patients undergoing chronic PD or HD for at least one year in the Nephrology Department of our hospital between December 26, 2024 to January 15, 2025. The study included 74 ESRD patients: 51 on HD and 23 on PD. Sleep quality was assessed using the Pittsburgh Sleep Quality Index (PSQI); a score ≥5 indicated poor sleep quality.

**Results:**

Poor sleep quality was observed in 56% of participants, consistent with previous research. No significant differences were found between HD and PD groups in overall sleep quality. Subjective sleep quality, sleep duration, efficiency, disturbances and use of sleep medication were similar in both groups. However, HD patients had significantly higher sleep latency and daytime dysfunction. Most patients with poor sleep quality were in the HD group.

**Conclusion:**

While dialysis modality did not significantly affect overall sleep quality, HD patients experienced more issues with sleep latency and daytime dysfunction. These findings underscore the need to address sleep disturbances in dialysis care.

## INTRODUCTION

Sleep, an important indicator of overall quality of life, is frequently disrupted in patients undergoing dialysis.^1^ It is estimated that between 43% and 80% of patients receiving continuous ambulatory peritoneal dialysis (PD) and approximately 45% to 85% of patients undergoing hemodialysis (HD), experience some form of sleep disturbance.^2^ The presence of accompanying diseases and the level of systemic inflammation often influence the presence and severity of these sleep disorders.^3^ A significant proportion of patients undergoing HD and PD therapy suffer from at least one sleep disorder.^4^ Common manifestations include trouble falling asleep, disturbances in maintaining sleep, waking up early in the morning, reduced sleep quality and shortened total sleep duration.^5^ The quality of life is negatively affected by sleep disturbances, leading to increased morbidity and decreased survival rates.^6^ Consequently, early diagnosis and interventions aimed at improving sleep quality are crucial for patients undergoing dialysis treatment.^7^

Studies evaluating sleep quality among patients receiving HD and PD have consistently demonstrated poor sleep quality in both groups.^8^ However, there is a need for further investigation into whether the different dialysis modalities, PD and HD, affect sleep quality in distinct ways in end-stage renal disease patients. Our aim was to assess the quality of sleep-in dialysis patients and compare the impact of PD and HD treatment modalities on sleep quality in this population.

## METHODOLOGY

In this observational, cross-sectional study in ESRD patients undergoing HD and PD, we compared the sleep quality with respect to the dialysis modality. The data were gathered from adult patients (>18 years of age) followed up in our Nephrology department between December 26, 2024 to January 15, 2025. The patients with the prior diagnosis of psychological disorders or mental impairment, those on sleep medicines and individuals unable to complete the questionnaire were excluded from the study. HD group consisted of subjects who have been on chronic HD program for at least one year period. The PD group included subjects who have been on chronic PD program for at least one year period. All participants had signed an informed consent form.

### Ethical approval:

The study was approved by Sancaktepe İlhan Varank Training Hospital, Department of Chest diseases ethics committee (389/25.12.2024; dated: December 26, 2024).

The same clinician has evaluated the patients individually and had explained the study. All patients were administered Turkish form of Pittsburgh Sleep Quality Index (PSQI)^9^,^10^ after being interviewed. It consists of a series of questions that evaluate sleep patterns, sleep disturbances and overall sleep quality. A total score of five or higher on the Pittsburgh Sleep Quality Index (PSQI) indicates poor sleep quality. The PSQI scores of the respondent so were compared in HD and PD patients. Continuous data were presented as mean ± standard deviation (SD). Sleep disturbances were assessed based on the mean PSQI scores. The t-test was applied to compare in age and PSQI scores between the HD and PD groups. The chi-square test was used to examine differences in gender and dialysis type between patients with poor and good sleep quality. Statistical analyses were performed using the Statistical Package for the Social Sciences (SPSS), version 19, with p-values less than 0.05 considered statistically significant.

## RESULTS

The study group consisted of 74 participants; 51 HD patients and 23 PD patients. The mean age was 53.93±12.11(28-80) years. Female/male / ratio was 36/38. The mean age was 52.75±11.34 in the HD group and 56.57±13.57 in the PD group (p=0.21). The gender distribution was similar across both groups, with male patients making up 51% of the HD group and 52% of the PD group. The PSQI results of the total study population and the two groups of patients undergoing dialysis are demonstrated in Table-I. PSQI scores were similar in HD and PD patients. Sleep quality concerning subjective sleep quality, sleep duration, habitual sleep efficiency, sleep disturbances and use of sleep medication were found to be similar in both groups. Sleep latency and daytime dysfunction were significantly higher in the HD group.

**Table-I:** Pittsburgh Sleep Quality Index in the study population and in dialysis groups.

	All participants	HD patients	PD patients	P value
Pittsburgh Sleep Quality Index	5.59±3.56	5.94±3.40	4.83±3.85	0.21
1-Subjective sleep quality	1.08±0.87	1. 06±0.83	1.13±0.96	0.74
2-Sleep latency	1.22±0.96	1.37±0.87	0.97±0.86	0.03
3-Sleep duration	0.80±1.09	0.78±1.02	0.83±1.26	0.88
4-Habitual sleep efficiency	0.39±0.75	0.39±0.80	0.39±0.65	0.99
5-Sleep disturbances	1.45±0.64	1.49±0.64	1.35±0.64	0.38
6-Use of sleep medication	0.12±0.46	0.12±0.47	0.13±0.45	0.91
7-Daytime dysfunction	0.50±0.78	0.67±0.84	0.13±0.45	0.005

Forty-two patients had PSQI score ≥5; 33 of them were HD patients and nine were PD patients (p=0.04). In this group male and female patients were nearly equal (22 and 20, respectively). This means, the majority of the patients with disturbed sleep quality were in the HD group. The time elapsed for the patient to fall asleep after going to bed was higher in HD group. More patients in the HD group have reported that their social activities were restricted due to poor sleep quality.

## DISCUSSION

Sleep disturbance is a common complaint among dialysis patients and has significant negative consequences for their health and well-being. While the prevalence of sleep disorders in the general population is reported to be between 20% and 30%, this rate increases dramatically to approximately 90% in patients undergoing dialysis.^11^ Sleep disturbances in this population can adversely affect the immune system and contribute to increased morbidity and mortality.^8^

In our study, we evaluated sleep disturbances in patients undergoing PD and HD, comparing the sleep quality between these two dialysis modalities to determine if one treatment type had a greater impact on sleep quality than the other. In our group, poor sleep quality was present in 56%. This result aligns with previous studies. Poor sleep quality has been reported to range from 40% to 90% in ESRD patients.^12^

Previous studies have suggested that sleep quality varies between genders; women reportedly experiencing worse quality of sleep.^13^ However, this current study has not shown significant correlation of gender and age with quality of sleep. This discrepancy may be attributed to variations in study design or patient demographics.

Several studies have compared sleep quality in PD and HD patients. One study reported that poor sleep quality was more frequent in HD patients than PD patients.^8^ Another study found that HD patients exhibited worse sleep quality across various sleep parameters.^11^ However, we found no difference of overall quality of sleep in the two dialysis groups. Despite this, we did find that HD patients had significantly higher sleep latency and greater daytime dysfunction, as indicated by the PSQI scoring system. These results suggest that, while overall sleep quality may not differ significantly between the two groups, HD patients experience specific challenges related to sleep initiation and daytime functioning.

Similarly, Holley et al. also found no association between dialysis modality and overall sleep quality.^14^ However, in a cohort study that diverged from our findings, HD patients were reported to have better sleep quality and overall quality of life compared to their PD counterparts.^3^ The differences in daytime dysfunction and sleep latency observed in our study may be attributed to better volume control in PD. Additionally, the regularity of dialysis treatment in PD patients, compared to the more variable nature of HD, may explain some of the differences observed in daytime activity levels.

Sleep disturbances can be attributed to various factors in patients with ESRD. Pruritus, a common and distressing symptom in HD patients, has also been identified as a major contributor to sleep problems. A previous multicenter cross-sectional study has examined the prevalence of chronic kidney disease-associated pruritus and its impact on sleep quality among HD patients in Pakistan. The findings indicate that pruritus is highly prevalent, affecting 74% of patients and is significantly linked to reduced sleep quality. The study also highlights that pruritus often goes unreported, leading to inadequate treatment and further deterioration in sleep patterns.^15^

Further supporting this, in a systematic review the impact of pruritus on the sleep quality of patients undergoing HD was investigated and a clear association was found between pruritus severity and sleep quality and quantity**,** reinforcing the importance and the need for effective management of addressing symptom management to improve overall patient well-being.^16^

Psychological distress, including anger and suicidal ideation, has also been shown to correlate with sleep disturbance. A study from Pakistan has investigated the relationship between anger, suicidal ideation and sleep disturbance in HD patients and have highlighted that emotional distress in HD patients can significantly compromise sleep, emphasizing the necessity for integrated psychological care within nephrology services.^17^

Zubair and Butt have examined the quality of sleep-in patients undergoing HD and have explored its relationship with psychiatric comorbidities and socio demographic characteristics**.** They have reported a high prevalence of poor sleep quality among HD patients, with a significant association with psychiatric disorders including anxiety and depression. Additionally, socio-demographic variables such as age, gender, low educational status and economic back ground, were found to negatively impact sleep quality in these patients.^18^

Another study has compared sleep quality between patients with chronic kidney disease not on HD and those undergoing HD. The findings indicate that sleep disturbances are prevalent in both groups, but patients on HD experience significantly poorer sleep quality compared to those with CKD. Factors such as physiological stress, metabolic fluctuations or psychosocial factors possibly contribute to these differences.^19^ Taken together; these findings emphasize the multifactorial nature of sleep disturbances in HD patients.

### Limitations:

One limitation of our study is the lack of polysomnography, which would provide a more comprehensive and objective assessment of sleep quality. Additionally, the limited size of the study population prevents the generalizability of the results. Another limitation is the potential for recall bias due to the self-reported nature of the PSQI, which may have influenced the accuracy of reported sleep quality.

## CONCLUSION

While no significant difference in overall quality of sleep was found comparing PD and HD patients in our study, certain components of sleep quality, such as latency and daytime dysfunction, were more problematic for HD patients. Considering various accompanying symptoms such as pruritis, socio-demographic factors or psychological problems may help mitigate sleep disturbances. Multidisciplinary interventions and integrated care approaches, including psychological support and lifestyle modifications are crucial to improving sleep quality, overall health outcomes and quality of life**.** Early identification and intervention can lead to substantial improvements in patient well-being and treatment efficacy**.**
